# Febuxostat provides renoprotection in patients with hyperuricemia or gout: a systematic review and meta-analysis of randomized controlled trials

**DOI:** 10.1080/07853890.2024.2332956

**Published:** 2024-05-13

**Authors:** Xiu Hong Yang, Bao Long Zhang, Yun Cheng, Shun Kun Fu, Hui Min Jin

**Affiliations:** a Department of Nephrology, Shanghai Pudong Hospital, Fudan University, Pudong Medical Center, Shanghai, China; b Department of Nephrology, Huadong Hospital, Fudan University, Shanghai, China; c The Institutes of Biomedical Sciences (IBS), Fudan University, Shanghai, China; d Department of Nephrology, The People’s Hospital of Wenshan Prefecture, Yunnan Province, China

**Keywords:** Meta-analysis, febuxostat, kidney events, eGFR

## Abstract

**Purpose:**

It is unknown whether febuxostat can delay the progression of kidney dysfunction and reduce kidney endpoint events. The aim was to evaluate the renoprotective effect of febuxostat in patients with hyperuricemia or gout by performing a meta-analysis of randomized controlled trials (RCTs).

**Methods:**

MEDLINE, Web of science, EMBASE, ClinicalTrials.gov, and the Cochrane Central Register for Randomized Controlled Trials were searched. The main outcomes included kidney events (serum creatinine doubling or progression to end-stage kidney disease or dialysis). The secondary outcomes were the rate of change in the estimated glomerular filtration rate (eGFR) and changes in the urine protein or urine albumin to creatinine ratio from baseline to the end of follow-up. We used random-effects models to calculate the pooled risk estimates and 95% CIs.

**Results:**

A total of 16 RCTs were included in the meta-analysis. In comparison with the control group, the patients who received febuxostat showed a reduced risk of kidney events (RR = 0.56, 95% CI 0.37–0.84, *p* = 0.006) and a slower decline in eGFR (WMD = 0.90 mL/min/1.73 m^2^, 95% CI 0.31–1.48, *p* = 0.003). The pooled results also revealed that febuxostat use reduced the urine albumin to creatinine ratio (SMD = −0.21, 95% CI −0.41 to −0.01, *p* = 0.042).

**Conclusion:**

Febuxostat use is associated with a reduced risk of kidney events and a slow decline in eGFR. In addition, the urine albumin to creatinine ratio decreased in febuxostat users. Accordingly, it is an effective drug for delaying the progression of kidney function deterioration in patients with gout.

**Systematic review registration:** PROSPERO CRD42021272591

## Graphical Abstract

**Figure:**
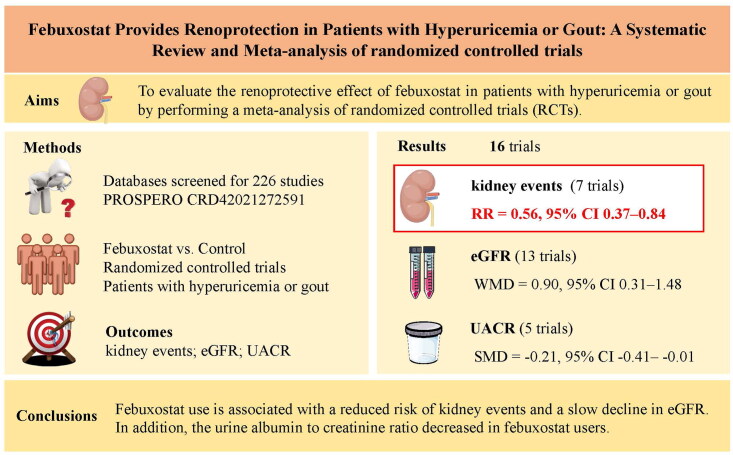

## Introduction

Febuxostat, a specific xanthine oxidase inhibitor, has been extensively used for the treatment of gout and hyperuricemia. In comparison with the previously used nonspecific xanthine oxidase inhibitor allopurinol, febuxostat is, mostly, hepatically metabolized, so having less dose adjustment procedures in kidney disease patients. However, some concerns have been recently identified in relation to an increased risk of cardiovascular disease (CVD) events and all-cause mortality [1]. In our previous meta-analysis of 20 randomized controlled trials (RCTs), febuxostat use was not associated with increased risks of all-cause mortality, death from CVD, or CVD events [2]. Accordingly, it was shown to be a safe drug for the treatment of gout. However, the renoprotective effect of urate-lowering therapy with febuxostat or allopurinol remains controversial. Pooled results from several meta-analyses showed contradictory findings for urate-lowering therapy, including inconclusive or nonsignificant changes in estimated glomerular filtration rate [eGFR] or unclear effectiveness in retarding the decrease in eGFR [3,4]. In a 2020 meta-analysis of 28 trials with 6458 participants, urate-lowering therapy did not improve clinical outcomes such as major adverse cardiovascular events, all-cause mortality, and kidney failure, and the findings showed insufficient evidence to support the effects of urate-lowering therapy in improving kidney outcomes [5]. Nevertheless, these meta-analyses had important defects, and their conclusions were obtained from a mixture of the results for febuxostat and allopurinol, yielding negative conclusions. The most recent meta-analysis, comprising three studies and encompassing a total of 145 patients, revealed that the use of febuxostat did not result in significant changes in eGFR [6]. However, the limited number of included articles and cases casts doubt on the reliability of these findings. More importantly, no hard kidney endpoint events (serum creatinine doubling or eGFR decline ≥30% from baseline or progression to end-stage kidney disease or dialysis) were summarized, only having pooled the changes of eGFR, a surrogate marker for kidney function. Two larger important RCTs in 2020 indicated that the urate-lowering effects of allopurinol did not slow the decline in eGFR and reduced hard kidney endpoints in chronic kidney disease and type 1 diabetes [7,8], though serum creatinine level was significantly lower in allopurinol treatment subjects with diabetes than that of the control group from a small sample size meta-analysis [9]. It is unknown whether febuxostat can delay the progression of kidney dysfunction and reduce kidney endpoint events. Therefore, the aim of this study is to conduct a meta-analysis from RCTs to confirm that febuxostat is effective in reduction of kidney events.

## Methods

We developed and followed a standard protocol according to the Preferred Reporting Items for Systematic reviews and Meta-Analyses statement. This study is registered with PROSPERO, number CRD42021272591.

### Search strategy and study selection

**Figure 1.**
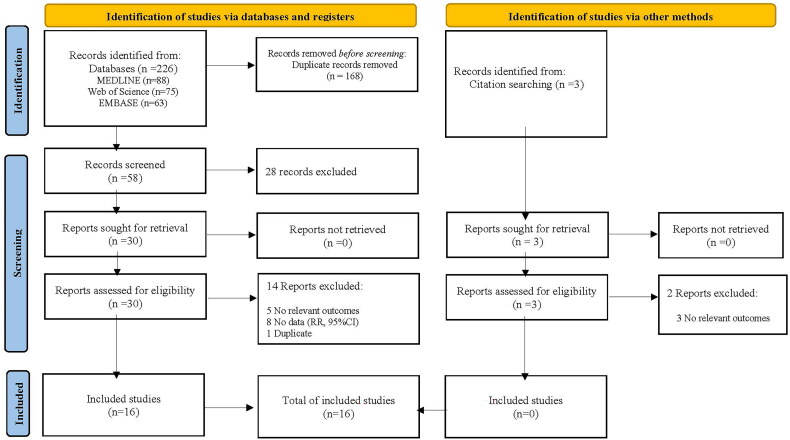
Study selection process (PRISMA 2020 flow diagram).

The relevant literature was searched in several databases, including MEDLINE (PubMed, Jan. 1, 1966 to Nov. 1, 2023), Web of Science, EMBASE (Jan. 1, 1966 to Nov.1, 2023), ClinicalTrials.gov, and Cochrane Central Register of Randomized Controlled Trials. The keywords were as follows: [(febuxostat) AND ((hyperuricemia) OR (uric acid) OR (gout)) AND RCT]. Manual searches of references cited by the identified original studies and relevant review articles were also performed and evaluated. All the studies in this meta-analysis were published in English. The detailed steps were presented in Figure 1 and Table S1.

### Inclusion and exclusion criteria

Studies that met the following criteria were included in our meta-analysis: (1) duration of not less than 8 weeks, (2) RCTs studies; (3) inclusion of kidney outcomes of febuxostat in comparison with a control group (placebo, allopurinol, or no treatment); and (4) availability of an outcome (kidney events, eGFR, and urine protein or urine albumin creatinine ratio). The main outcome is kidney events included doubling of serum creatinine concentration, eGFR decline ≥30%, end-stage renal disease (ESRD), and initiation of dialysis therapy. The secondary outcomes are the changes of eGFR (a surrogate marker of kidney function), urine protein or urine albumin creatinine ratio before and after therapy.

Studies were excluded if they met any of the following criteria: (1) comparison of outcomes between febuxostat and control groups were not reported, (2) no description of eGFR, kidney events, (3) different publications analyzing the same population or duplicates and (4) study population after kidney transplantation.

### Data collection

Two researchers (XHY and BLZ) performed the search and reviewed the results. Data were collected by all authors and independently extracted by the two researchers, who reviewed all the study characteristics (i.e., the first author’s surname, year of publication, study design, sample, follow-up, and outcomes). Cohen’s kappa was performed to examine the differences between the two authors in the selection (Cohen’s kappa = 0.97, strong agreement) [10]. Any disagreement in data extraction was resolved through a discussion among these researchers in consultation with the other authors (CY, JHM and SKF).

### Assessment of heterogeneity

Heterogeneity was evaluated using Galbraith plots and I^2^ statistics. Studies with I^2^ values less than 50% were considered to be non-heterogeneous, whereas studies with I^2^ > 50% were considered to be heterogeneous. For heterogeneity values >50%, meta-regression was performed to explore its potential source. The studies of this meta-analysis were analyzed using a random-effects model (the Hartung–Knapp/Sidik–Jonkman random-effects method) [11].

### Quality assessment

The grading of recommendations, assessment, development, and evaluation (GRADE) system (https://gdt.gradepro.org/app/) was used to evaluate the quality of the evidence. The evaluation included the study design, risk of bias, inconsistency, indirectness, imprecision, and other considerations. Four quality levels were developed: ‘very low’, ‘low’, ‘moderate’, and ‘high’.

### Risk of bias assessment

Cochrane’s Risk of Bias 2 (RoB 2) tool was used to assess the risk of bias in individual studies. Five domains of the RCTs were assessed: randomization process, deviations from intended interventions, missing outcome data, measurement of the outcome, and selection of the reported result. The methodologies were classified as high risk, some concerns, and low risk. The overall ROB for each trial was determined based on the highest risk in any domain.

### Outcome measures

The primary outcome was a kidney event. As secondary outcomes, we assessed the rate of changes in eGFR (mL/min/1.73 m^2^) and changes in the urine protein or urine albumin to creatinine ratio from baseline to the end of follow-up.

### Statistical analyses

The data were analyzed using STATA version 17.0 (StataCorp, TX, USA). The risk ratio (RR) and risk difference (RD) for the association between febuxostat and kidney events were either calculated or extracted from individual studies. Subgroup analyses were also performed to evaluate the effects of the age, sex, various disease conditions (such as the presence or absence of diabetes, hypertension, asymptomatic hyperuricemia or gout), and geographical region on the risk of kidney events. The weighted mean difference (WMD) was used to pool the change-from-baseline values for eGFR, which was reported using the same scale of mea­surement in all studies. We also analyzed the urine protein/albumin levels of the studies included in this meta-analysis. The standardized mean difference (SMD) was used to pool results from all studies that reported untransformed changes in the urine protein or urine albumin to creatinine ratio, which can be used to compare studies that report continuous outcomes using different scales. We also conducted a sensitivity analysis in which each study was extracted to evaluate the effect of the study on the estimate. The Egger’s test was used to examine the presence of a publication bias. The statistical significance for all analyses was set at *p* < 0.05.

## Results

### Study flow and study characteristics

The decision process for inclusion is shown in Figure 1. Overall, 16 studies were included [12–27]. Tables 1 and 2 show the characteristics of the 16 included studies on febuxostat. Seven studies recorded kidney events, 13 evaluated eGFR, and six evaluated urine protein or urine albumin to creatinine ratio values.

**Table 1.** Characteristics of 16 studies associated with kidney events, eGFR, and urinary protein or urine albumin creatinine ratio in hyperuricemia population.

Study	N	Age (years)	Sex (male%)	Pre-existing condition	Study type	Follow up	Control group (mg/d)	Febuxostat group(mg/d)	Outcomes
Goldfarb DS et al. 2013 [12]	66	47.4 ± 10.3	87.9	Hyperuricemia	Double-blind, multicenter, RCT	6 months	Allopurinol 200 or 300; placebo	80	Urinary protein
Sezai A et al. 2013 [13]	140	67.4 ± 10.3	82.1	Hyperuricemia in cardiac surgery patients	RCT	6 months	Allopurinol 200 or 300	40 or 60	eGFR, urinary albumin,
Sircar D et al. 2015 [14]	93	56.22 ± 10.87	71.0	Hyperuricemia in CKD stage 3–4	Double-blind, RCT	6 months	Placebo	40	Kidney events eGFR
Tanaka K et al. 2015 [15]	40	70.1 ± 9.5	87.5	Hyperuricemia in CKD 3	Open-label, parallel-group, RCT	12 weeks	Allopurinol 100 or 50	10 or 40	eGFR, urinary protein
Tani S et al.2015 [16]	60	67.0 ± 12.0	88.0	Hyperuricemic patients	RCT	6 months	Without UA lowering agents	10	eGFR,
Beddhu S et al. 2016 [17]	80	68.0 ± 10.0	65.0	Hyperuricemic patients with diabetic nephropathy	Double-blind, RCT	24 weeks	Placebo	80	eGFR
Saag KG et al. 2016 [18]	96	65.7 ± 10.6	80.2	Gout patients with moderate-to-severe renal impairment	Multicenter, double-blind, RCT	12 months	Placebo	60, 40 or 80	Kidney events eGFR
Gunawardhana L et al.2018 [19]	189	61.3 ± 10.1	71.0	Gout and moderate renal impairment (CKD stage 3)	Multicenter, double-blind, RCT	3 months	Placebo	40, 80	Kidney events
Kimura K et al.2018 [20]	441	65.3 ± 11.8	77.3	Hyperuricemia in CKD stage 3	Double-blind, RCT	108 weeks	Placebo	10, 20, or 40	Kidney events eGFR
Mukri MNA et al.2018 [21]	93	64.0 ± 10.0	53.8	Diabetic nephropathy hyperuricemia in CKD stage 3–4	prospective open-label, RCT	6 months	Without UA lowering agents	40	eGFR, Urine albumin creatinine ratio
Kojima S et al.2019 [22]	1070	75.7 ± 6.6	69.1	Hyperuricemia in CKD stage 3	Multicentre, prospective, RCT	36 months	Allopurinol 100	10, 20, or 40	Kidney events eGFR,
Wen H et al.2020 [23]	38	58.73 ± 11.5	86.8	CKD3 diabetic nephropathy	RCT	24 weeks	Without UA lowering agents	20, 40 or 60	eGFR, urinary protein
Yang N et al. 2022 [24]	120	18–80	73.3	Hyperuricemia in HKD stage 2–3	RCT	6 months	Allopurinol 200	20	Kidney events eGFR
Kohagura K et al.2023 [25]	95	64.6 ± 14.9	66.3	Hyperuricemia in CKD stage 3	RCT	52 weeks	Benzbromarone 25	20	eGFR urinary protein
Nana N et al.2023 [26]	84	68.0 ± 15.6	66.7	Hyperuricemia in CKD stage 3–4	RCT	8weeks	Without UA lowering agents	40	eGFR
Yang HT et al. 2023 [27]	100	20–75	72.8	Hyperuricemia in CKD stage 3–4	Multicenter, RCT	12 months	Without UA lowering agents	40	Kidney events

CKD, chronic kidney disease; eGFR, estimated glomerular filtration rate; RCT, randomized controlled trial; UA: uric acid kidney events-doubling of serum creatinine concentration, eGFR decline ≥30% from baseline, initiation of dialysis therapy, and ESRD (end stage renal disease).

**Table 2.** Baseline of uric acid, GFR, and urinary protein or urine albumin creatinine ratio of included studies.

	Baseline uric acid (mg/dL)		Baseline GFR (mL/min/1.73 m^2^)			Baseline proteinuria or ACR		
Study	Control group	Febuxostat group	Measure	Control group	Febuxostat group	Measure	Control group	Febuxostat group
Goldfarb DS et al. 2013[12]	6.3 ± 1.49	6.2 ± 1.63	eGFR	≥30	≥30	urinary protein (mg/24 h)	125.9 ± 79.0	199.9 ± 308.3
Sezai A et al. 2013[13]	≥8	≥8	eGFR	48.5 ± 16.6	47.5 ± 17.3	urinary albumin (mg/24h)	143.8 ± 272.2	144.5 ± 371.9
Sircar D et al. 2015[14]	8.2 ± 1.1	9.0 ± 2.0	eGFR	32.6 ± 11.4	31.5 ± 13.6	/	/	/
Tanaka K et al. 2015[15]	8.18 ± 1.11	7.75 ± 0.84	eGFR	47.4 ± 11.0	41.8 ± 12.0	UACR (g/gCr)	0.43 ± 0.71	0.91 ± 1.44
Tani S et al.2015[16]	7.48 ± 0.97	7.66 ± 1.10	eGFR	68.8 ± 19.9	62.2 ± 16.0	/	/	/
Beddhu S et al. 2016[17]	422 ± 71 (umol/L)	426 ± 89 (umol/L)	eGFR	53.5 ± 17.2	52.2 ± 15.3	/	/	/
Saag KG et al. 2016[18]	10.8 ± 1.96	10.4 ± 1.43	eGFR	29.31 ± 4.77	34.14 ± 4.84	/	/	/
Gunawardhana L et al.2018[19]	9.7 ± 1.2	9.8 ± 1.4	eGFR	47.3 ± 9.4	46.0 ± 8.3	/	/	/
Kimura K et al.2018[20]	7.8 ± 0.9	7.8 ± 0.9	eGFR	44.9 ± 9.7	45.2 ± 9.5	UACR (mg/gCr)	120.5(17.2–517.0)	124.0(19.1–525.0)
Mukri MNA et al.2018[21]	539.5 ± 103.4 (umol/L)	537.3 ± 70.6 (umol/L)	eGFR	28.2(19.8)	26.2(14.3)	UPCI (g/mmol)	0.17(0.33)	0.13(0.29)
Kojima S et al.2019[22]	7.50 ± 1.03	7.54 ± 1.06	eGFR	55.35 ± 15.16	54.62 ± 14.11	UACR (g/gCr)	0.086(0.044–0.170)	0.082(0.043–0.165)
Wen H et al.2020[23]	423.4 ± 51.2 (umol/L)	447.5 ± 83.6 (umol/L)	eGFR	46.8 ± 9.0	45.3 ± 10.6	urinary protein (g/24 h)	4.02 ± 2.67	4.15 ± 2.58
Yang N et al. 2022[24]	9.19 ± 1.71	9.06 ± 1.51	eGFR	55.1 ± 15.9	54.3 ± 15.2	urinary protein (g/24 h)	0.819 ± 0.588	0.809 ± 0.687
Kohagura K et al.2023[25]	8.50 ± 1.13	8.20 ± 0.95	eGFR	44.3 ± 8.2	44.4 ± 7.8	UACR (mg/gCr)	75.7(16.2–202.0)	36.4(13.6–338.0)
Nana N et al.2023[26]	8.39 ± 1.37	8.90 ± 1.35	eGFR	31.82 ± 14.35	31.22 ± 10.23	UACR (mg/gCr)	229.3(48–762)	331.4(14.1–1534)
Yang HT et al. 2023[27]	7.92 ± 0.89	8.71 ± 1.72	eGFR	32.6 ± 8.7	29.9 ± 10.8	urinary protein (g/24 h)	0.98(0.66–2.08)	0.86(0.26–1.92)

eGFR, estimated glomerular filtration rate; UACR, urinary albumin-creatinine ratio; UPCI, urine protein creatinine index. Values for continuous variables, data are expressed as mean ± SD; non-normally distributed data, data are expressed as median (IQR) or median (quartile 1–quartile 3); SD, standard deviation; IQR, interquartile range.

### Association between febuxostat and kidney events

**Figure 2.**
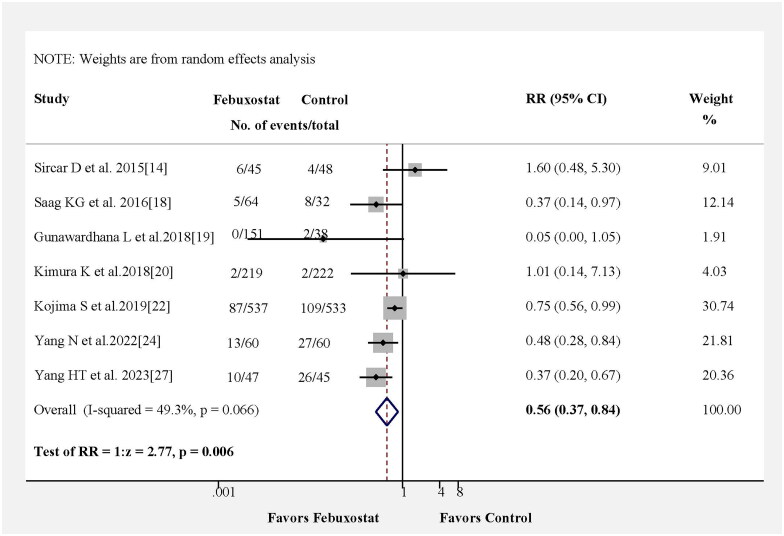
Risk ratio (RR) for kidney events associated with febuxostat from pooled studies. Kidney events included the doubling of the serum creatinine concentration, eGFR decline ≥30% from baseline, end-stage renal disease (ESRD), and initiation of dialysis therapy.

In Figure 2, we reassessed the benefits of febuxostat treatment in kidney events in seven studies. The pooled results from seven studies indicated that in comparison with the control group, those receiving febuxostat showed a reduced risk of kidney events (RR = 0.56, 95% CI 0.37–0.84, *p* = 0.006) with no heterogeneity among the studies (Figure S1A). Kidney events also were observed for the pooled RD estimation, where the RD for patients receiving febuxostat was −0.09 (95% CI: −0.18–0.00, *p* = 0.04) (Figure S2).

### Subgroup analysis of relative risk for kidney events

The age, sex, various disease conditions (such as the presence or absence of diabetes, hypertension, asymptomatic hyperuricemia or gout), and geographical region were potential confounders related to kidney events. As shown in Figure S3, the estimated RR suggested that the febuxostat group was associated with a decreased risk of kidney events in patients aged <75 years compared to the control group (RR = 0.58, 95% CI 0.39–0.85). Similarly, the estimated RRs also suggested that febuxostat group decreases the risk of kidney events regardless of asymptomatic hyperuricemia or gout (RR = 0.65, 95% CI 0.52–0.81; RR = 0.30, 95% CI 0.12–0.76, respectively). Furthermore, the estimated RRs indicate that the febuxostat group exhibited a decreased risk of kidney events in both the Asia and USA regions (RR = 0.62, 95% CI 0.50–0.77 and RR = 0.74, 95% CI 0.60–0.96, respectively) compared with the control regions. Sex, diabetes, and hypertension appeared to have no effect on kidney events.

### Association between febuxostat and eGFR

**Figure 3.**
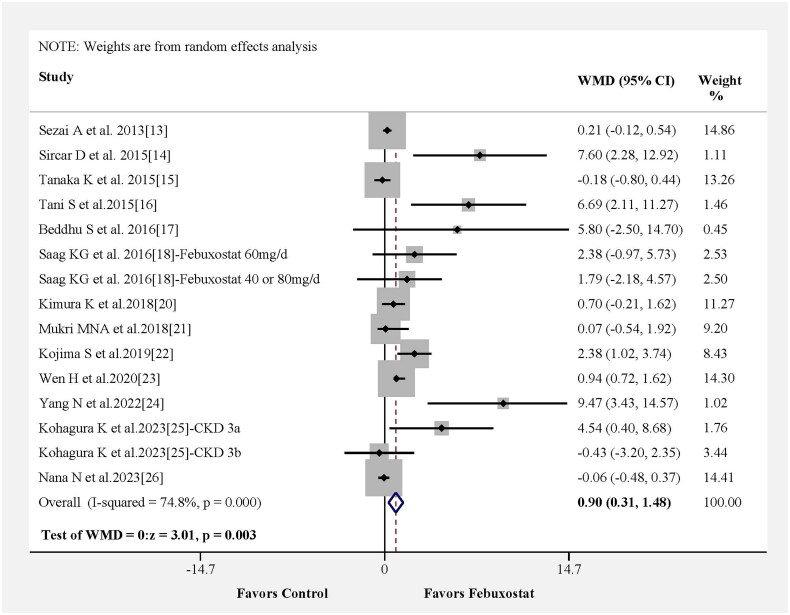
Mean difference (WMD) for eGFR associated with febuxostat from pooled studies. CKD3a: stage 3a of chronic kidney disease; CKD3b: stage 3b of chronic kidney disease.

In Figure 3, we investigated the effect of febuxostat on eGFR in 13 studies. Likewise, the change in WMD for eGFR was statistically significant, and pooled results from 13 studies showed that the febuxostat group had a higher eGFR than the control group (WMD = 0.90, 95% CI 0.31–1.48, *p* = 0.003) with moderate heterogeneity among the studies (Figure S1B). The results of the meta-regression analysis indicated that variables such as participant age, medication usage in the control group and study time were not significant contributors to heterogeneity (Figure S4A–C). Notably, follow-up time was identified as the principal source of heterogeneity (Figure S4D).

### Association of febuxostat with urine protein or urine albumin to creatinine ratio

**Figure 4.**
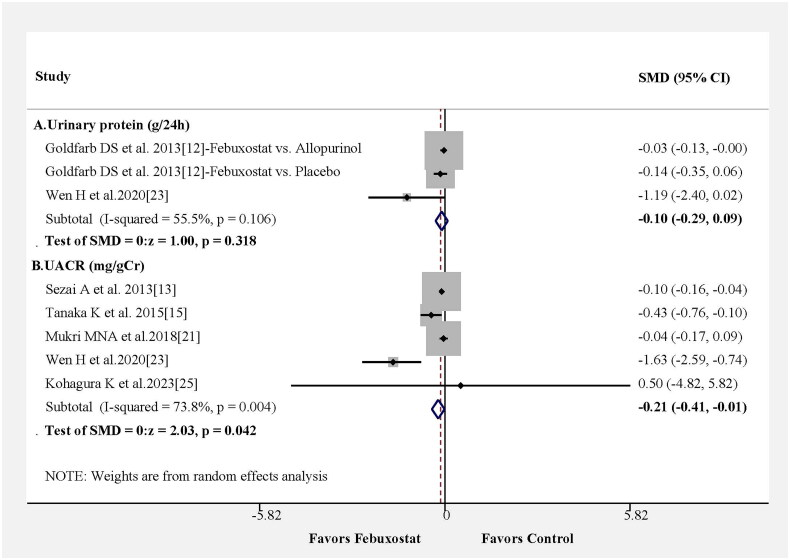
Changes in the urine protein or urine albumin to creatinine ratio in patients following febuxostat use compared with changes in the control group. Changes are expressed as the standardized mean difference (SMD). (A) Urinary protein (g/24h); (B) UACR (mg/gCr).

We also reassessed the benefits of febuxostat treatment in the urine protein or urine albumin to creatinine ratio in six studies. Urinary protein excretion (g/24h) was not significant difference between febuxostat and control group from only three results in two papers (Figure 4A). The changes in the SMD for urine albumin creatinine ratio were statistically significant, and the pooled results from five studies showed that the febuxostat group had a lower urine albumin to creatinine ratio than the control group, as shown in Figure 4B (SMD = −0.21; 95% CI −0.41– −0.01, *p* = 0.042) with moderate heterogeneity among the studies (Figure S1C).

### Sensitivity analysis and publication bias

The sensitivity analysis revealed that the exclusion of any individual study from the meta-analysis did not alter the overall conclusions. Consequently, no publication bias was found in the pooled studies (kidney events, *p* = 0.438; eGFR, *p* = 0.303). We also used the funnel plot to evaluate the publication bias for the outcomes (Figure S5).

### Risk of bias assessment and quality of evidence assessment

The risk assessment for any bias of RCTs was analyzed with the Cochrane’s Risk of Bias 2 (RoB 2) tool and presented in Figure S6. The GRADE system was used to assess the quality of the evidence. The evaluation results are shown in Table S2. In summary, the quality of evidence was rated as high for kidney events and moderate for eGFR and urine protein or urine albumin to creatinine ratio.

## Discussion

In this meta-analysis, our pooled results demonstrated that febuxostat use was associated with a significantly reduced risk of hard kidney endpoints and a slow decrease in eGFR, a surrogate marker, in comparison with allopurinol or placebo in patients with hyperuricemia or gout. Notably, the results of our meta-analysis were not consistent with those of other meta-analyses [3–6,9]. Previous meta-analyses primarily evaluated urate-lowering therapy either with febuxostat or allopurinol, and the mixed results obtained with febuxostat and allopurinol masked the actual effects of febuxostat. In a study by Liu X. et al. who conducted a network meta-analysis using seven RCTs on febuxostat vs. placebo or other drugs, the pooled results indicated that febuxostat did not exert superior effects on improving eGFR over placebo [4]. There are important flaws in those meta-analyses. First, no trials on febuxostat use and kidney outcome were included, and several important trials were missed. Second, the assessment of renal function progression did not use hard kidney endpoints, instead using only the change of eGFR, a surrogate marker. Therefore, the pooled results in those meta-analyses are not credible. Two high-quality RCTs [7,8] indicated that allopurinol did not slow the decline in eGFR or reduce hard kidney endpoints. A previously published meta-analysis on six RCTs comparing febuxostat with placebo concluded that febuxostat may slow the progression of CKD irrespective of baseline renal function; however, the progression of kidney function was only evaluated by determining the surrogate marker eGFR, and hard kidney endpoints were not evaluated. More importantly, the meta-analysis showed significant heterogeneity (I^2^ = 96.6% at 6 months and I^2^ = 98% at the end of the study) [28]. Therefore, this conclusion is unconvincing. Another earlier updated systematic review and meta-analysis in 2017 showed that xanthine oxidase inhibitors reduced the risk of the combined endpoint of progression to ESRD as compared with the controls from only three RCTs [29]. However, this conclusion was based on three allopurinol vs. control trials with very small sample sizes, and an insufficient statistical power was met. In our meta-analysis, our pooled results from the change in the eGFR was 0.9 ml/min/1.73 m^2^, probably being of little clinical significance for clinicians; however, it is significant from a statistical perspective. This result is consistent with the concomitant hard kidney endpoint result.

Many *in vivo* and *in vitro* studies have indicated that febuxostat can protect against kidney injury [30–34]. In acute ischemia/reperfusion renal injury in mice, febuxostat can block the degradation pathway of adenine nucleotides, promote ATP recovery, and exert renoprotective effects in the postischemic kidney [30]. In other studies, febuxostat has been shown to exert an anti-inflammatory action and protect against diabetic nephropathy development in KK-Ay obese diabetic mice [31], reduce ER stress through the upregulation of SIRT1-AMPK-HO-1/thioredoxin expression [32], inhibit TGFβ1-induced epithelial–mesenchymal transition *via* downregulation of USAG-1 expression in Madin–Darby canine kidney cells *in vitro* [33], and slow the development of nephropathy in experimental type 2 diabetes *via* the reduction of uric acid, renal oxidative stress, and inhibition of profibrotic signaling [34]. Oxidative stress is most likely a key mediator and is implicated in the progression and deterioration of chronic kidney disease. The prospective, block-randomized, double-blinded, and placebo-controlled study showed that febuxostat significantly decreased the serum malondialdehyde (MDA) and significantly increased the serum superoxide dismutase, and a positive correlation was observed between the change in serum asymmetric dimethylarginine (ADMA) levels and MDA levels in hemodialysis patients with endothelial dysfunction [35,36].

However, the potential association between febuxostat use and increased CVD risk and mortality has remained a concern. On February 21, 2019, the FDA reiterated that febuxostat use was associated with more cardiovascular deaths than allopurinol [37]. In a meta-analysis of 20 RCTs in 2021, our pooled results indicated that febuxostat use was not associated with increased risks of all-cause mortality, death from CVD, or CVD events. Accordingly, it is a safe drug for the treatment of gout [2].

This meta-analysis had several potential limitations. First, high-quality meta-analyses with data from RCTs should be conducted; although the papers included in our meta-analysis were RCTs, there are relatively few RCTs, and insufficient numbers of patients were included. Future larger multicenter trials including hard kidney endpoints are needed to confirm the results. Second, there was substantial heterogeneity in the changes in eGFR (I^2^ = 74.8%), meta-regression showed that great heterogeneity was probably due to the differences in follow up times (as shown in Figure S4D). However, the pooled results of the changes in eGFR are consistent with the results for hard kidney endpoints in the evaluation of the renoprotection provided by febuxostat.

In conclusion, this meta-analysis indicates that febuxostat use is associated with reduced risks of kidney events and a slower decline in eGFR. In addition, the urine albumin to creatinine ratio also decreased in febuxostat users. Thus, febuxostat may be an effective drug for delaying the progression of kidney function deterioration in patients with gout or hyperuricemia. More RCTs designed with hard kidney endpoints, other than surrogate marker-change of eGFR, is needed to further verify this results in future.

## Supplementary Material

Supplemental Material

Supplemental Material

## Data Availability

The dataset(s) supporting the conclusions of this article is (are) included within the article.
