# Supplementary material for: Febuxostat provides renoprotection in patients with hyperuricemia or gout: a systematic review and meta-analysis of randomized controlled trials
Source: Ann Med. 2024 May 13;56(1):2332956. doi: 10.1080/07853890.2024.2332956 (PMC11095284; doi:10.1080/07853890.2024.2332956)
Supplement: Supplemental Material [file IANN_A_2332956_SM8752.zip › graphic/Table.docx]

Table1. Characteristics of 16 studies associated with kidney events, eGFR, and urinary protein or urine albumin creatinine ratio in hyperuricemia population

| **Study** | **N** | **Age**  **(years)** | **Sex**  **(male%)** | **Pre-existing condition** | **Study type** | **Follow up** | **Control group (mg/d)** | **Febuxostat group(mg/d)** | **Outcomes** |
| --- | --- | --- | --- | --- | --- | --- | --- | --- | --- |
| Goldfarb DS et al. 2013[12] | 66 | 47.4±10.3 | 87.9 | hyperuricemia | double-blind, multicenter, RCT | 6 months | Allopurinol 200 or 300;  placebo | 80 | urinary protein |
| Sezai A et al. 2013[13] | 140 | 67.4±10.3 | 82.1 | hyperuricemia in cardiac surgery  Patients | RCT | 6 months | Allopurinol 200 or 300 | 40 or 60 | eGFR,  urinary albumin, |
| Sircar D et al. 2015[14] | 93 | 56.22±10.87 | 71.0 | hyperuricemia in CKD stage 3-4 | double-blind, RCT | 6 months | placebo | 40 | kidney events  eGFR |
| Tanaka K et al. 2015[15] | 40 | 70.1±9.5 | 87.5 | hyperuricemia in CKD 3 | open-label, parallel-group, RCT | 12 weeks | Allopurinol 100 or 50 | 10 or 40 | eGFR,  urinary protein |
| Tani S et al.2015[16] | 60 | 67.0±12.0 | 88.0 | hyperuricemic patients | RCT | 6 months | without UA lowering agents | 10 | eGFR, |
| Beddhu S et al. 2016[17] | 80 | 68.0±10.0 | 65.0 | hyperuricemic Patients  with diabetic nephropathy | double-blind, RCT | 24 weeks | placebo | 80 | eGFR |
| Saag KG et al. 2016[18] | 96 | 65.7±10.6 | 80.2 | Gout Patients  With Moderate-to-Severe Renal Impairment | multicenter,  double-blind,  RCT | 12 months | placebo | 60, 40 or 80 | kidney events  eGFR |
| Gunawardhana L et al.2018[19] | 189 | 61.3±10.1 | 71.0 | gout and moderate renal impairment (CKD stage 3) | multicenter,  double-blind,  RCT | 3 months | placebo | 40, 80 | kidney events |
| Kimura K et al.2018[20] | 441 | 65.3±11.8 | 77.3 | hyperuricemia in CKD stage 3 | double-blind, RCT | 108 weeks | placebo | 10, 20, or 40 | kidney events  eGFR |
| Mukri MNA et al.2018[21] | 93 | 64.0 ±10.0 | 53.8 | diabetic nephropathy hyperuricemia in CKD stage 3-4 | prospective open-label, RCT | 6 months | without UA lowering agents | 40 | eGFR,  Urine albumin creatinine ratio |
| Kojima S et al.2019[22] | 1070 | 75.7±6.6 | 69.1 | hyperuricemia in CKD stage 3 | multicentre, prospective, RCT | 36 months | Allopurinol 100 | 10, 20, or 40 | kidney events  eGFR, |
| Wen H et al.2020[23] | 38 | 58.73±11.5 | 86.8 | CKD3 diabetic nephropathy | RCT | 24 weeks | without UA lowering agents | 20, 40 or 60 | eGFR,  urinary protein |
| Yang N et al. 2022[24] | 120 | 18-80 | 73.3 | hyperuricemia in CKD stage 2-3 | RCT | 6 months | Allopurinol 200 | 20 | kidney events  eGFR |
| Kohagura K et al.2023[25] | 95 | 64.6±14.9 | 66.3 | hyperuricemia in CKD stage 3 | RCT | 52 weeks | benzbromarone 25 | 20 | eGFR  urinary protein |
| Nana N et al.2023[26] | 84 | 68.0±15.6 | 66.7 | hyperuricemia in CKD stage 3-4 | RCT | 8weeks | without UA lowering agents | 40 | eGFR |
| Yang HT et al. 2023[27] | 100 | 20-75 | 72.8 | hyperuricemia in CKD stage 3-4 | multicenter, RCT | 12 months | without UA lowering agents | 40 | kidney events |

CKD, chronic kidney disease; eGFR, estimated glomerular filtration rate; RCT, randomized controlled trial; UA: uric acid

kidney events- doubling of serum creatinine concentration, eGFR decline ≥30% from baseline, initiation of dialysis therapy, and ESRD (end stage renal disease)

Table2. Baseline of uric acid, GFR, and urinary protein or urine albumin creatinine ratio of included studies.

| **Study** | **Baseline uric acid (mg/dL)** | |  | **Baseline GFR (mL/min/1.73 m^2^)** | | |  | **Baseline proteinuria or ACR** | | |
| --- | --- | --- | --- | --- | --- | --- | --- | --- | --- | --- |
|  | **Control group** | **Febuxostat group** |  | **Measure** | **Control group** | **Febuxostat group** |  | **Measure** | **Control group** | **Febuxostat group** |
| Goldfarb DS et al. 2013[12] | 6.3±1.49 | 6.2±1.63 |  | eGFR | ≥30 | ≥30 |  | urinary protein (mg/24 h) | 125.9±79.0 | 199.9±308.3 |
| Sezai A et al. 2013[13] | ≥8 | ≥8 |  | eGFR | 48.5±16.6 | 47.5±17.3 |  | urinary albumin  (mg/24h) | 143.8±272.2 | 144.5±371.9 |
| Sircar D et al. 2015[14] | 8.2±1.1 | 9.0±2.0 |  | eGFR | 32.6±11.4 | 31.5±13.6 |  | / | / | / |
| Tanaka K et al. 2015[15] | 8.18±1.11 | 7.75±0.84 |  | eGFR | 47.4±11.0 | 41.8±12.0 |  | UACR (g/gCr) | 0.43±0.71 | 0.91±1.44 |
| Tani S et al.2015[16] | 7.48±0.97 | 7.66±1.10 |  | eGFR | 68.8±19.9 | 62.2±16.0 |  | / | / | / |
| Beddhu S et al. 2016[17] | 422±71  (umol/L) | 426±89 (umol/L) |  | eGFR | 53.5±17.2 | 52.2±15.3 |  | / | / | / |
| Saag KG et al. 2016[18] | 10.8±1.96 | 10.4±1.43 |  | eGFR | 29.31±4.77 | 34.14±4.84 |  | / | / | / |
| Gunawardhana L et al.2018[19] | 9.7±1.2 | 9.8±1.4 |  | eGFR | 47.3±9.4 | 46.0±8.3 |  | / | / | / |
| Kimura K et al.2018[20] | 7.8±0.9 | 7.8±0.9 |  | eGFR | 44.9±9.7 | 45.2±9.5 |  | UACR(mg/gCr) | 120.5(17.2-517.0) | 124.0(19.1-525.0) |
| Mukri MNA et al.2018[21] | 539.5±103.4 (umol/L) | 537.3±70.6 (umol/L) |  | eGFR | 28.2(19.8) | 26.2(14.3) |  | UPCI(g/mmol) | 0.17(0.33) | 0.13(0.29) |
| Kojima S et al.2019[22] | 7.50±1.03 | 7.54±1.06 |  | eGFR | 55.35±15.16 | 54.62±14.11 |  | UACR (g/gCr) | 0.086(0.044-0.170) | 0.082(0.043-0.165) |
| Wen H et al.2020[23] | 423.4±51.2 (umol/L) | 447.5±83.6 (umol/L) |  | eGFR | 46.8±9.0 | 45.3±10.6 |  | urinary protein (g/24 h) | 4.02±2.67 | 4.15±2.58 |
| Yang N et al. 2022[24] | 9.19±1.71 | 9.06±1.51 |  | eGFR | 55.1±15.9 | 54.3±15.2 |  | urinary protein (g/24 h) | 0.819±0.588 | 0.809±0.687 |
| Kohagura K et al.2023[25] | 8.50±1.13 | 8.20±0.95 |  | eGFR | 44.3±8.2 | 44.4±7.8 |  | UACR(mg/gCr) | 75.7(16.2-202.0) | 36.4(13.6-338.0) |
| Nana N et al.2023[26] | 8.39±1.37 | 8.90±1.35 |  | eGFR | 31.82±14.35 | 31.22±10.23 |  | UACR(mg/gCr) | 229.3(48-762) | 331.4(14.1-1534) |
| Yang HT et al. 2023[27] | 7.92±0.89 | 8.71±1.72 |  | eGFR | 32.6±8.7 | 29.9±10.8 |  | urinary protein (g/24 h) | 0.98(0.66-2.08) | 0.86(0.26-1.92) |

eGFR, estimated glomerular filtration rate; UACR, urinary albumin-creatinine ratio; UPCI, urine protein creatinine index. Values for continuous variables, data are expressed as mean ± SD; non-normally distributed data, data are expressed as median (IQR) or median (quartile 1-quartile 3); SD, standard deviation; IQR, interquartile range.
