# Supplementary figures and images for: Febuxostat provides renoprotection in patients with hyperuricemia or gout: a systematic review and meta-analysis of randomized controlled trials
Source: Ann Med. 2024 May 13;56(1):2332956. doi: 10.1080/07853890.2024.2332956 (PMC11095284; doi:10.1080/07853890.2024.2332956)

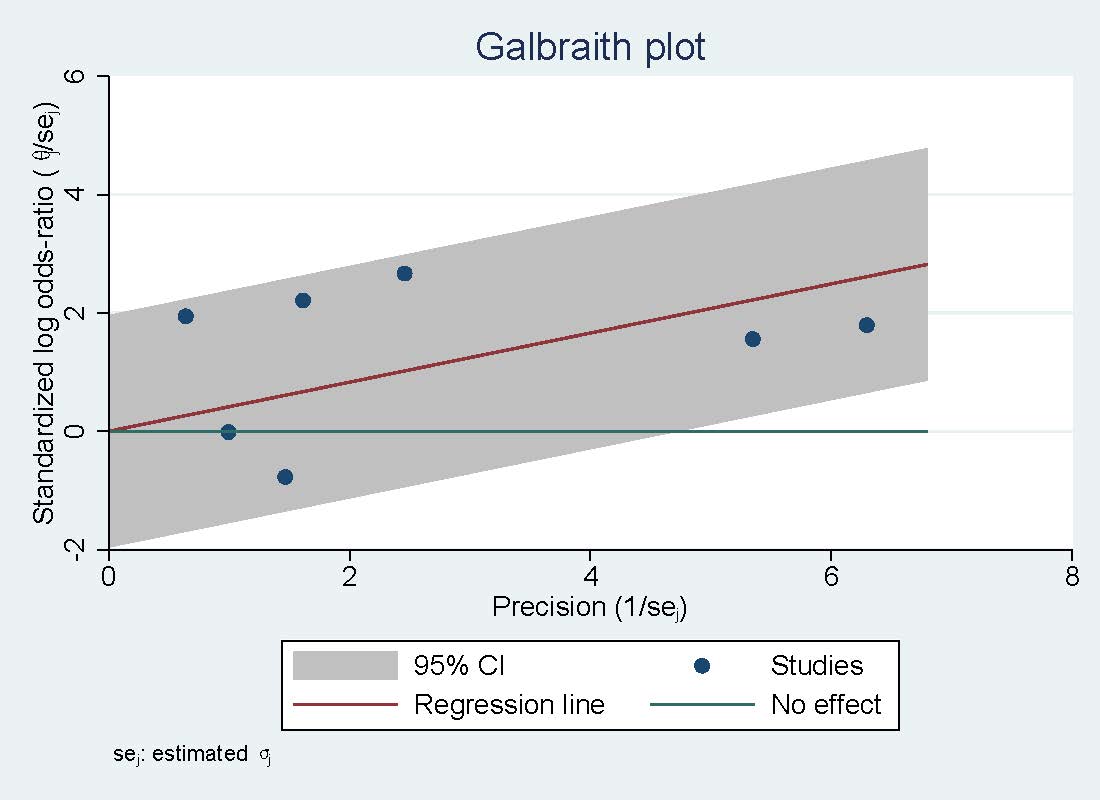

Supplement: Supplemental Material [file IANN_A_2332956_SM8752.zip › graphic/FigureS1A.jpg]

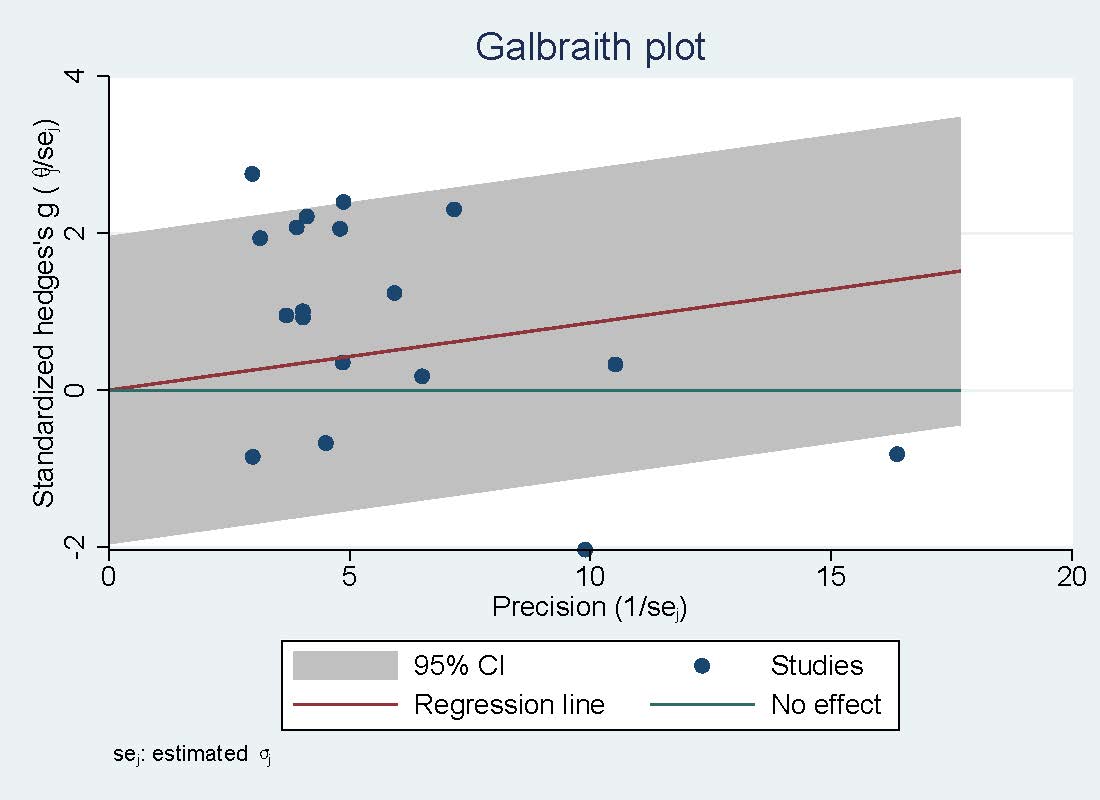

Supplement: Supplemental Material [file IANN_A_2332956_SM8752.zip › graphic/FigureS1B.jpg]

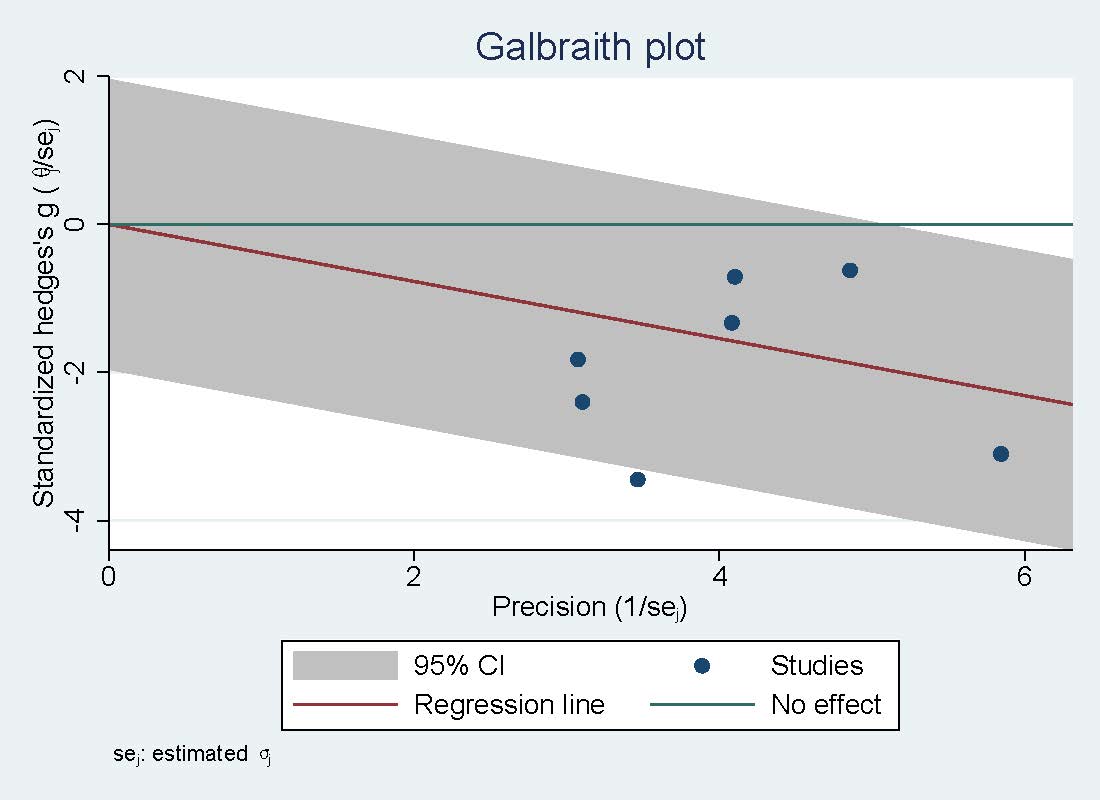

Supplement: Supplemental Material [file IANN_A_2332956_SM8752.zip › graphic/FigureS1C.jpg]

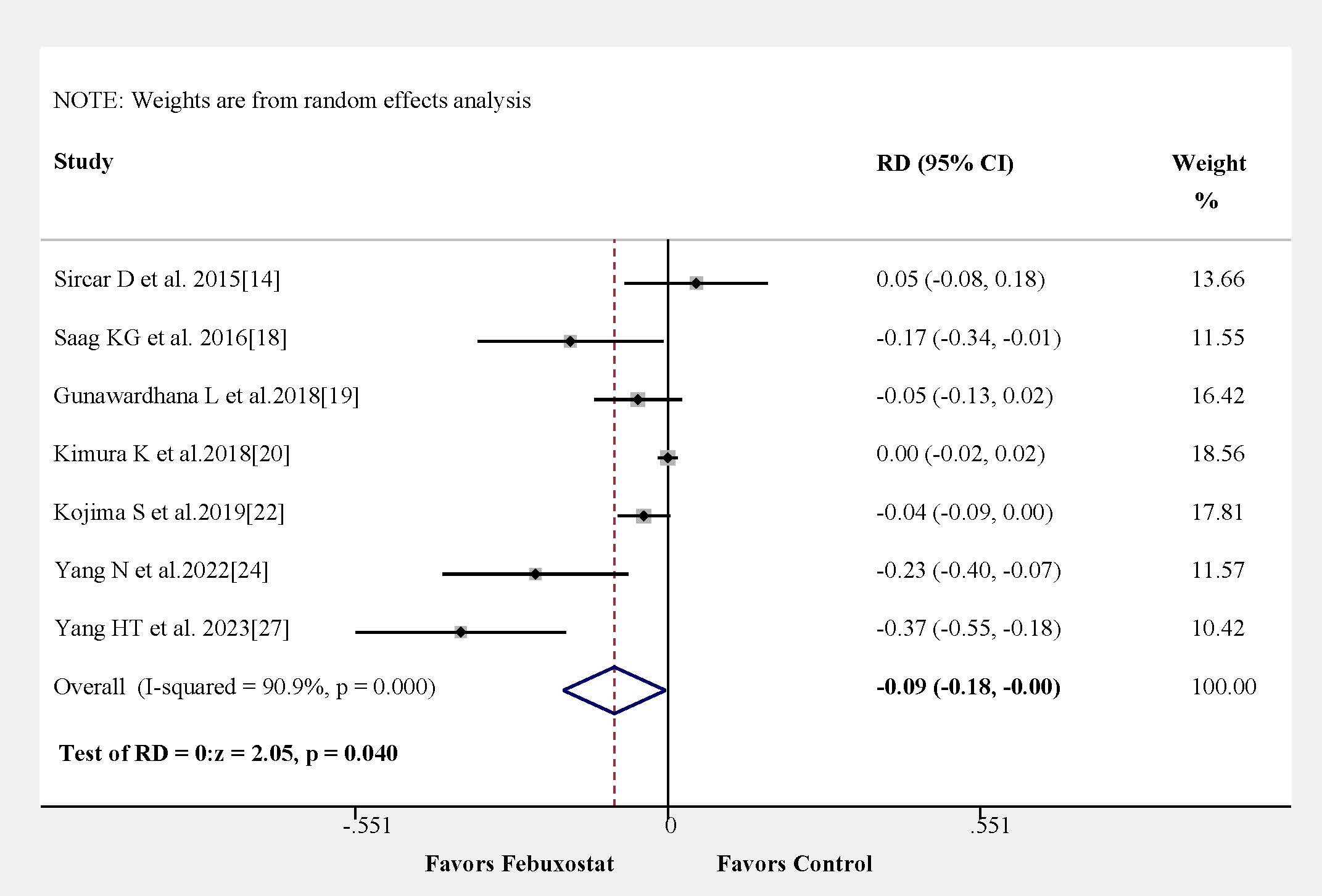

Supplement: Supplemental Material [file IANN_A_2332956_SM8752.zip › graphic/FigureS2.jpg]

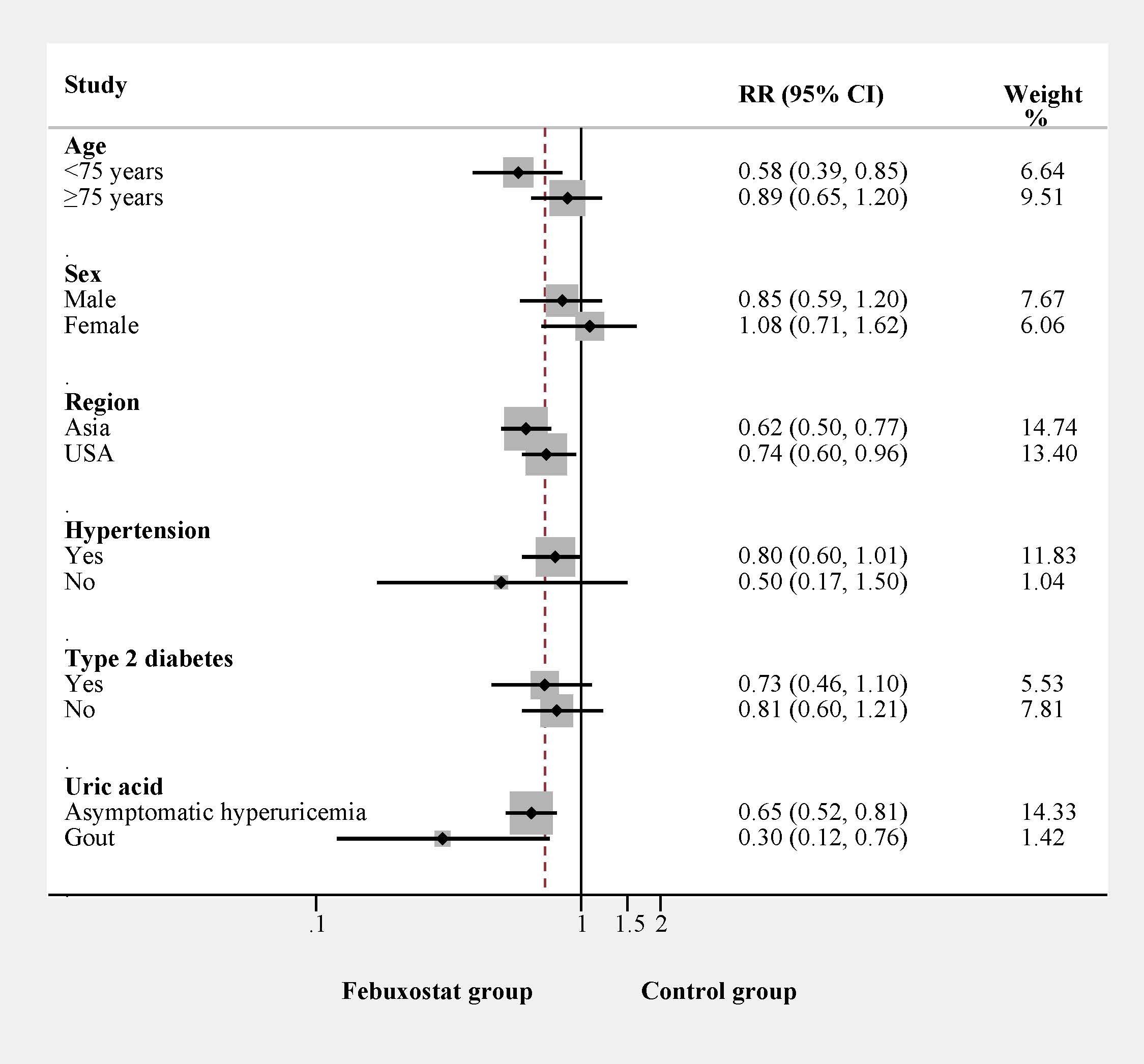

Supplement: Supplemental Material [file IANN_A_2332956_SM8752.zip › graphic/FigureS3.jpg]

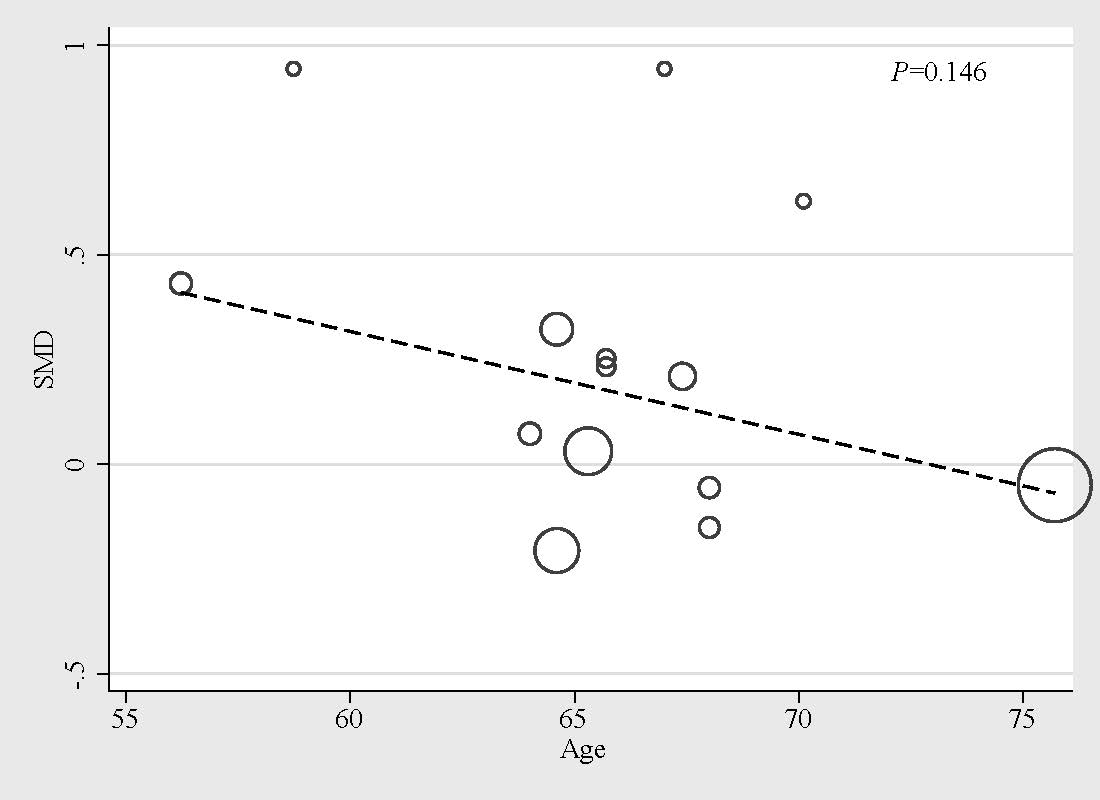

Supplement: Supplemental Material [file IANN_A_2332956_SM8752.zip › graphic/FigureS4A.jpg]

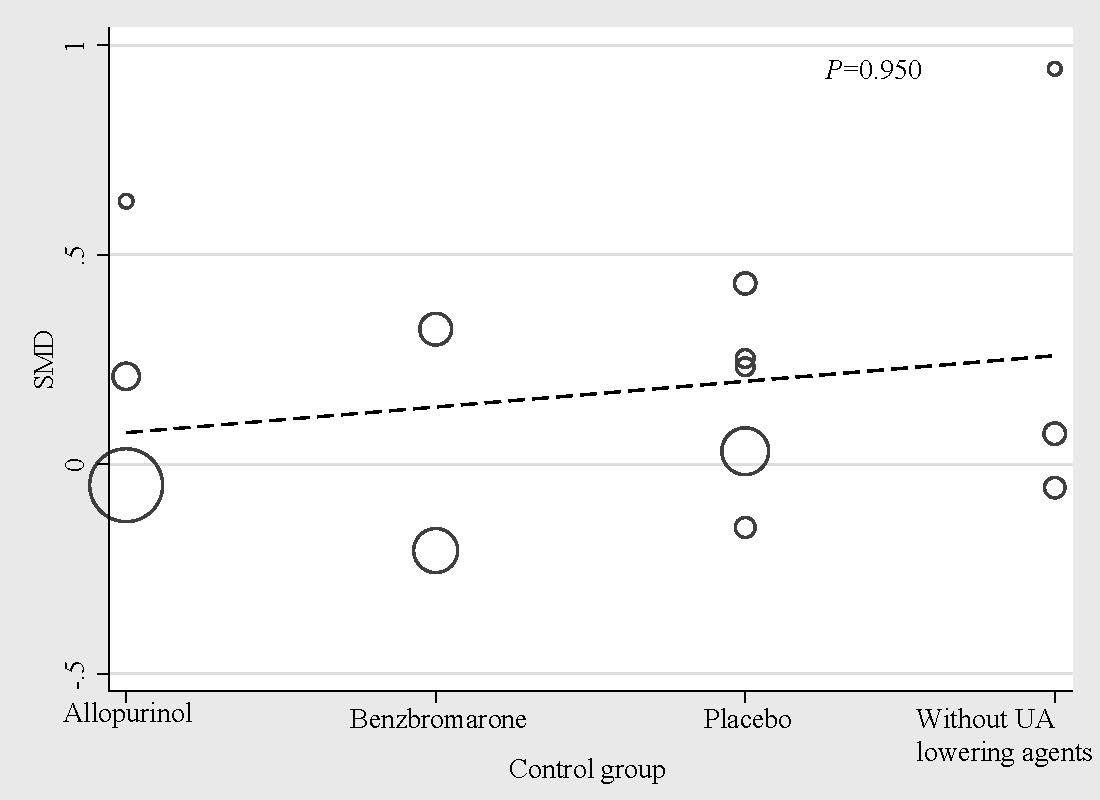

Supplement: Supplemental Material [file IANN_A_2332956_SM8752.zip › graphic/FigureS4B.jpg]

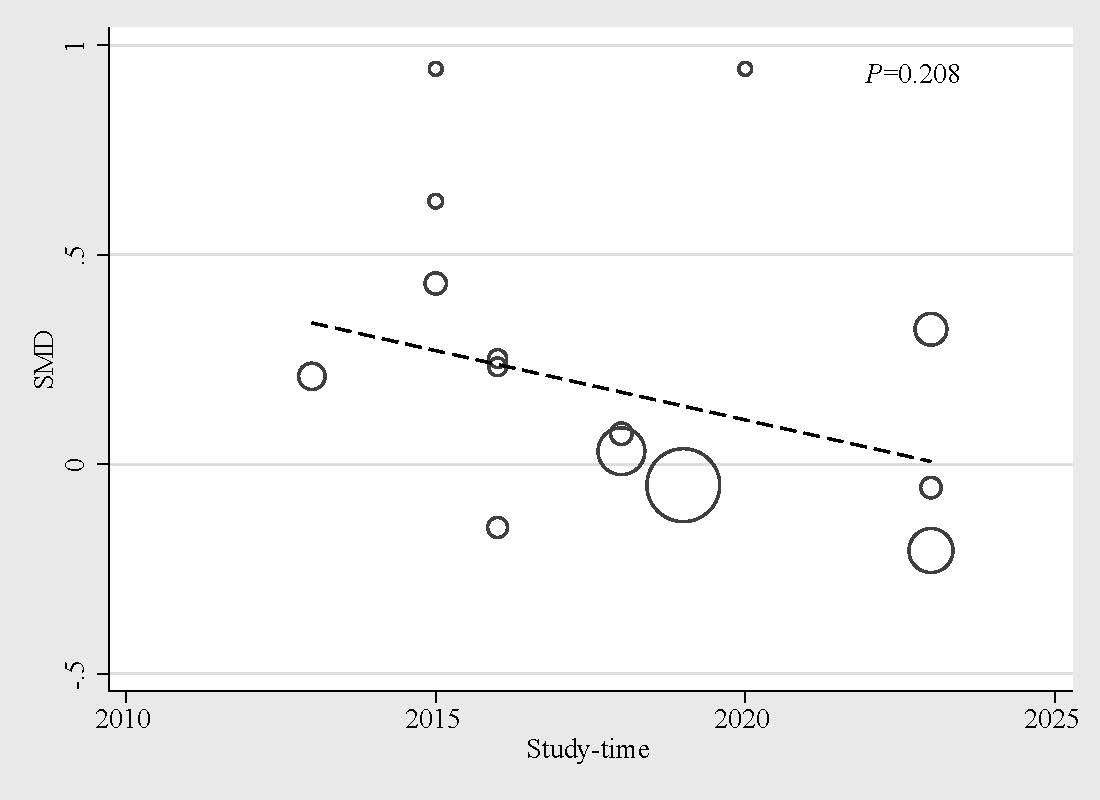

Supplement: Supplemental Material [file IANN_A_2332956_SM8752.zip › graphic/FigureS4C.jpg]

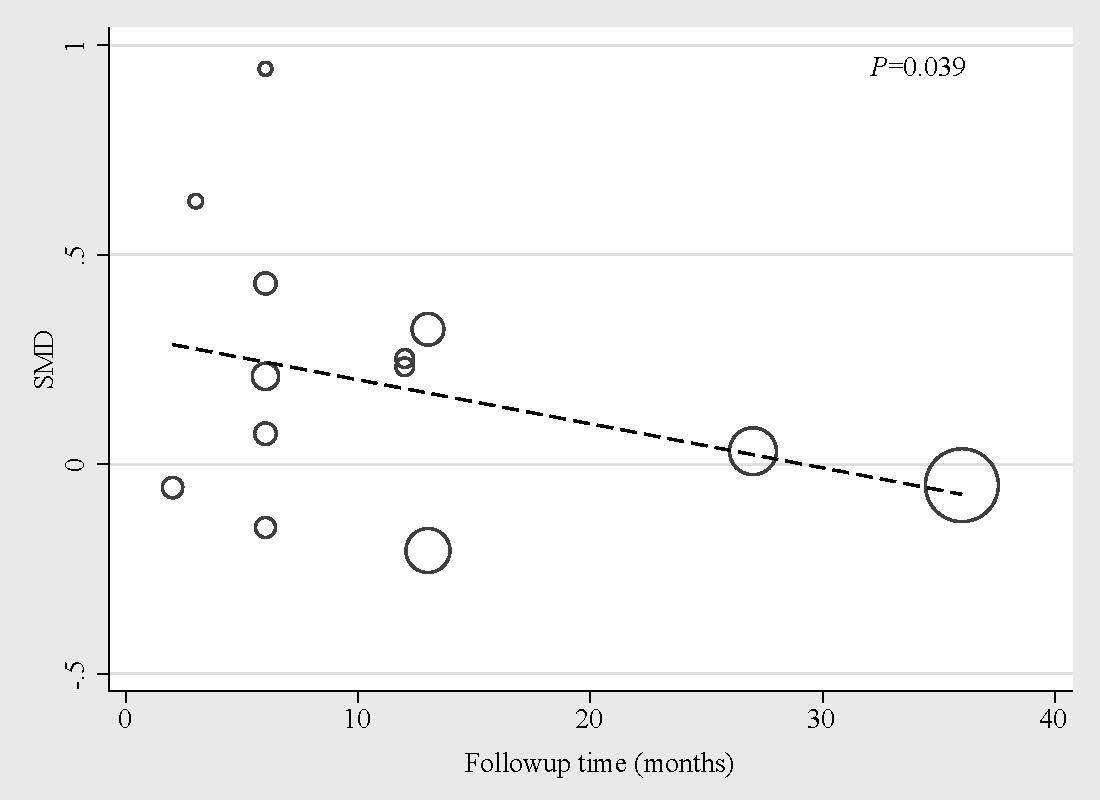

Supplement: Supplemental Material [file IANN_A_2332956_SM8752.zip › graphic/FigureS4D.jpg]

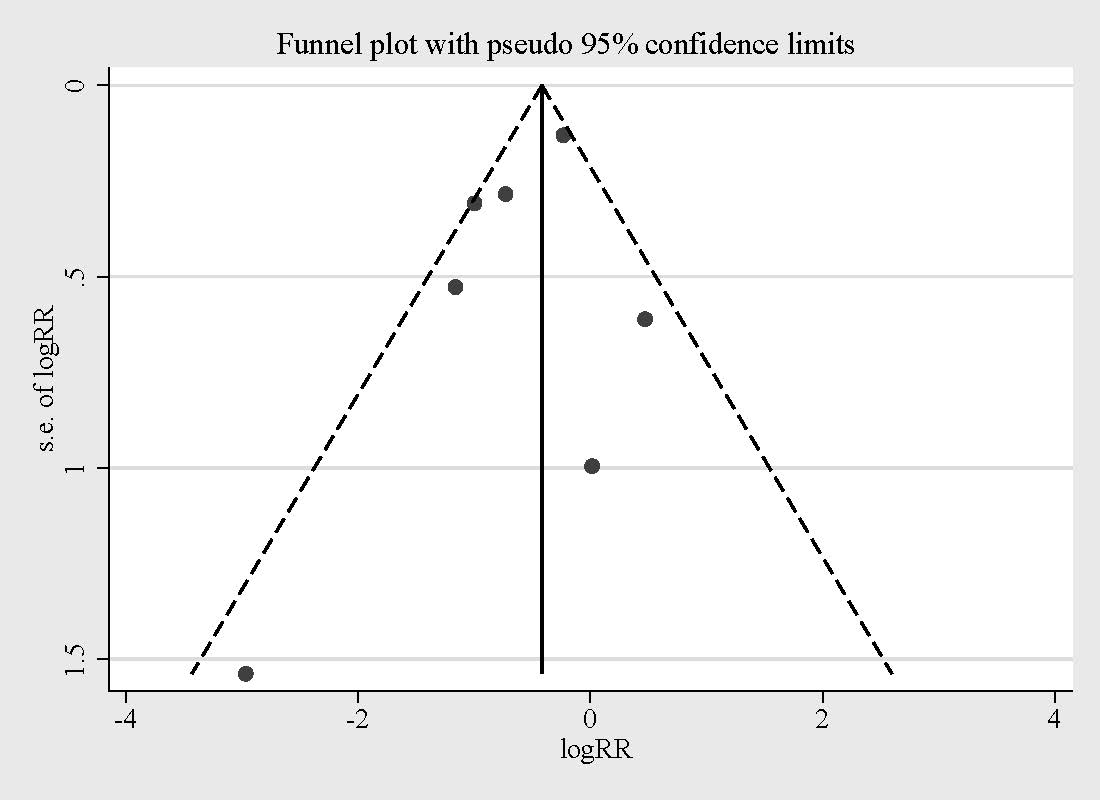

Supplement: Supplemental Material [file IANN_A_2332956_SM8752.zip › graphic/FigureS5A.jpg]

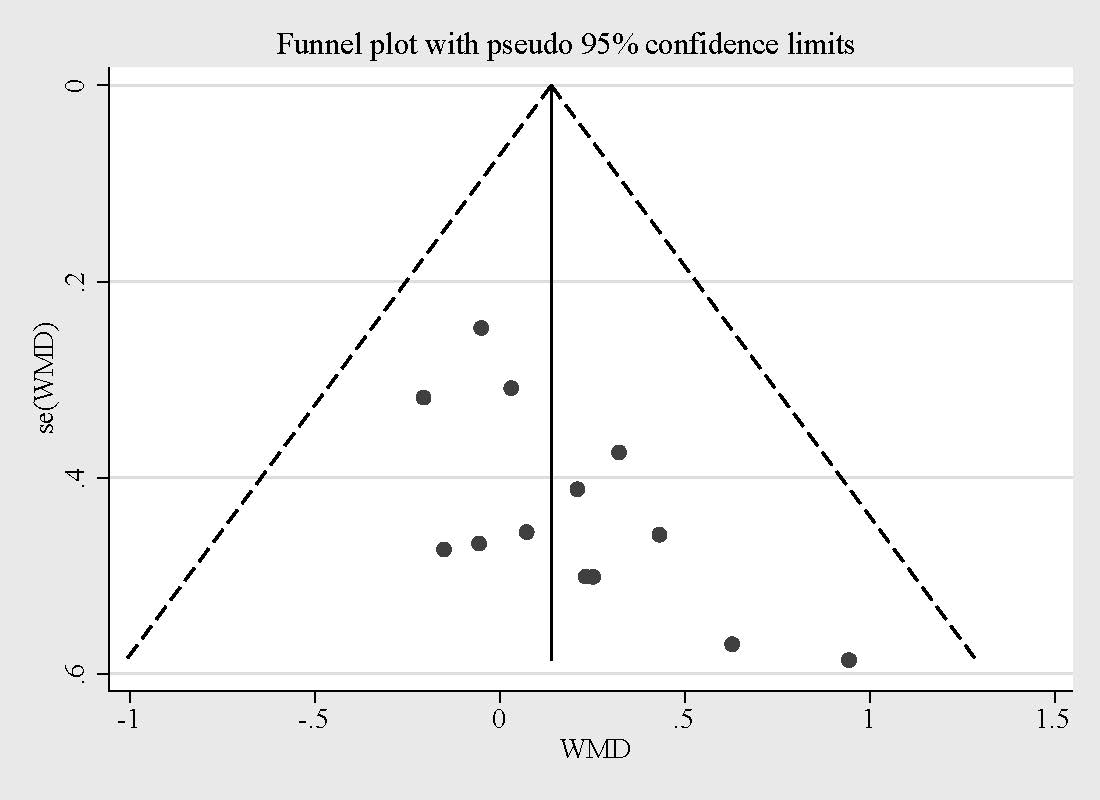

Supplement: Supplemental Material [file IANN_A_2332956_SM8752.zip › graphic/FigureS5B.jpg]

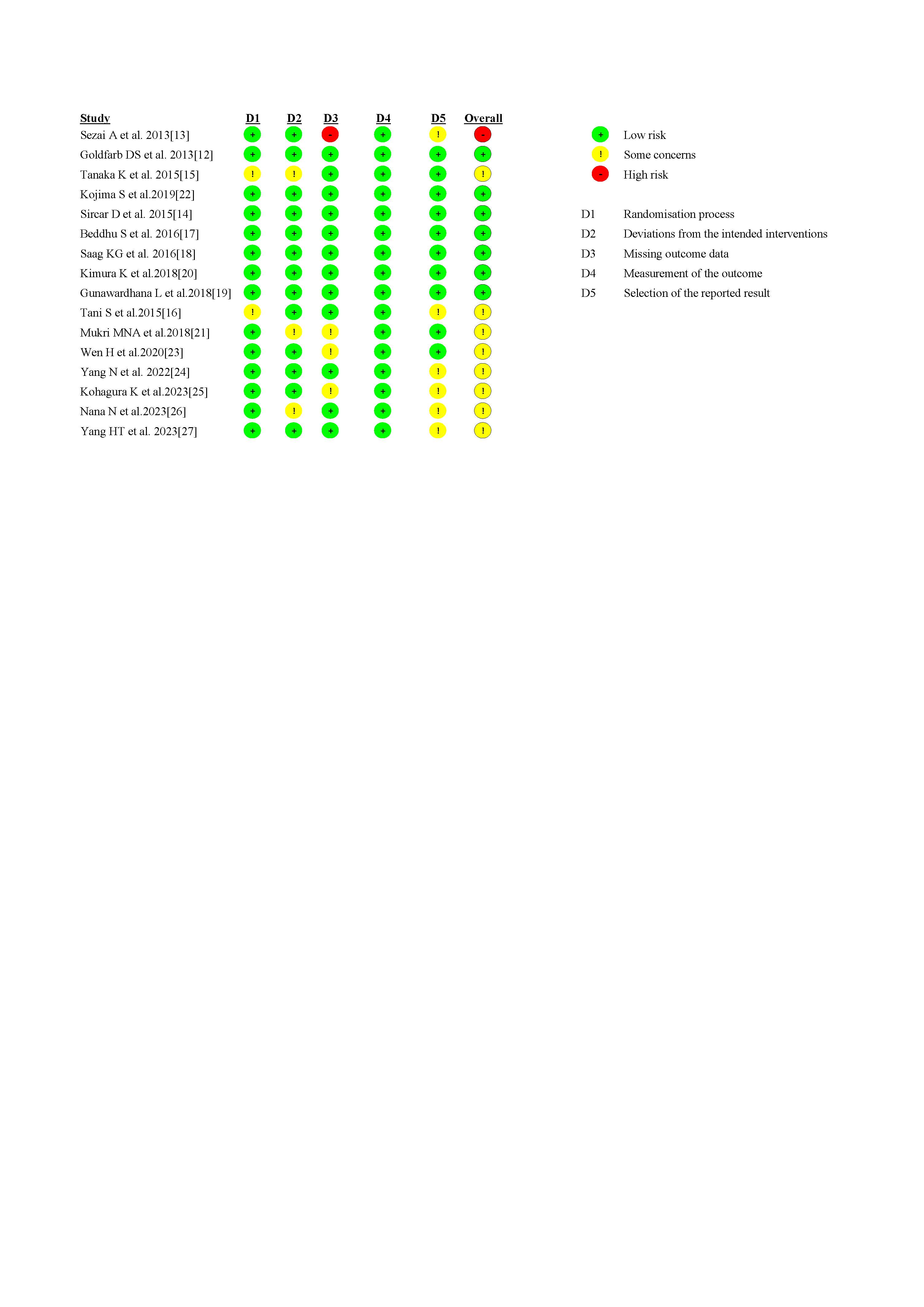

Supplement: Supplemental Material [file IANN_A_2332956_SM8752.zip › graphic/FigureS6A.jpg]

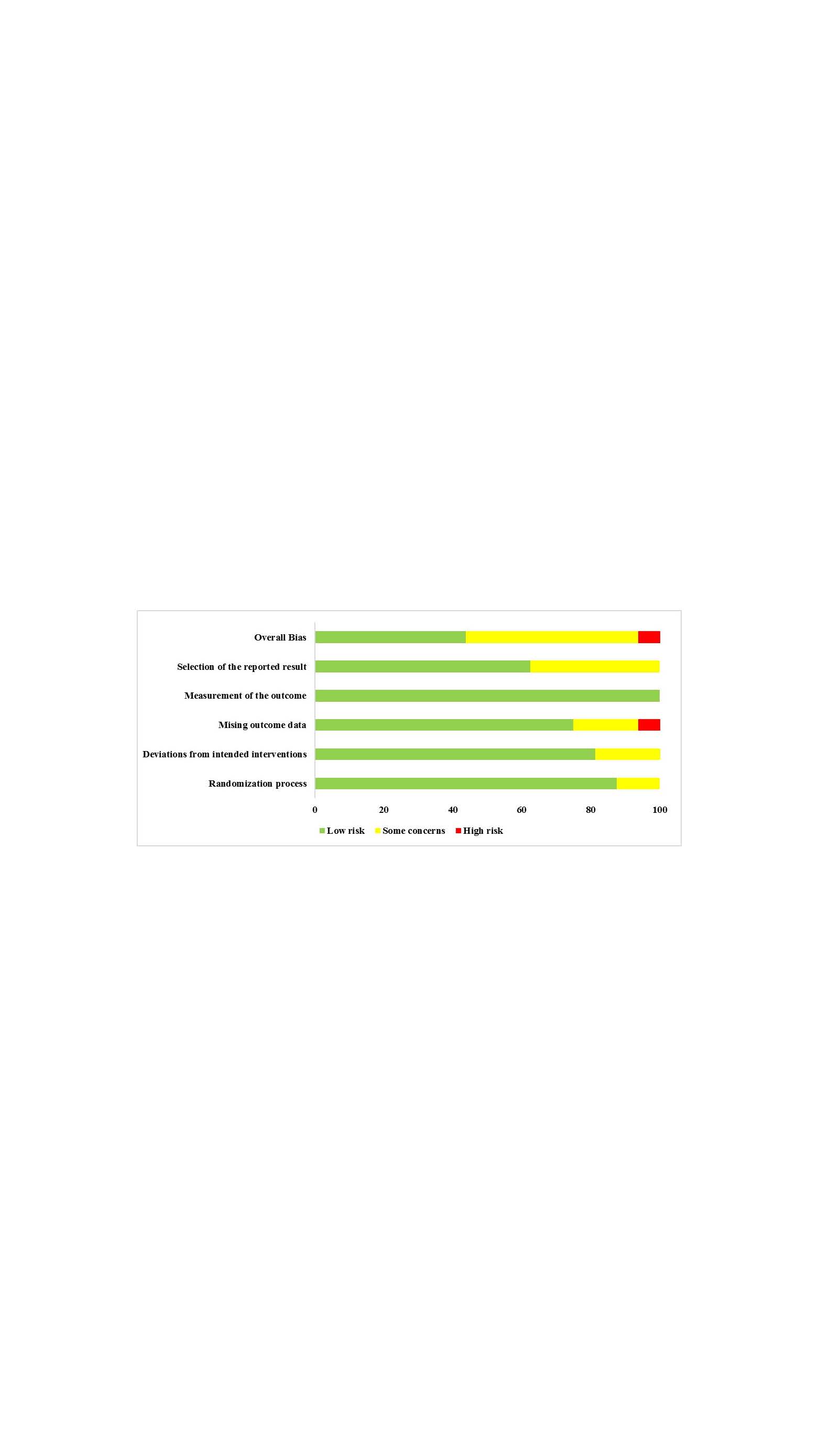

Supplement: Supplemental Material [file IANN_A_2332956_SM8752.zip › graphic/FigureS6B.jpg]

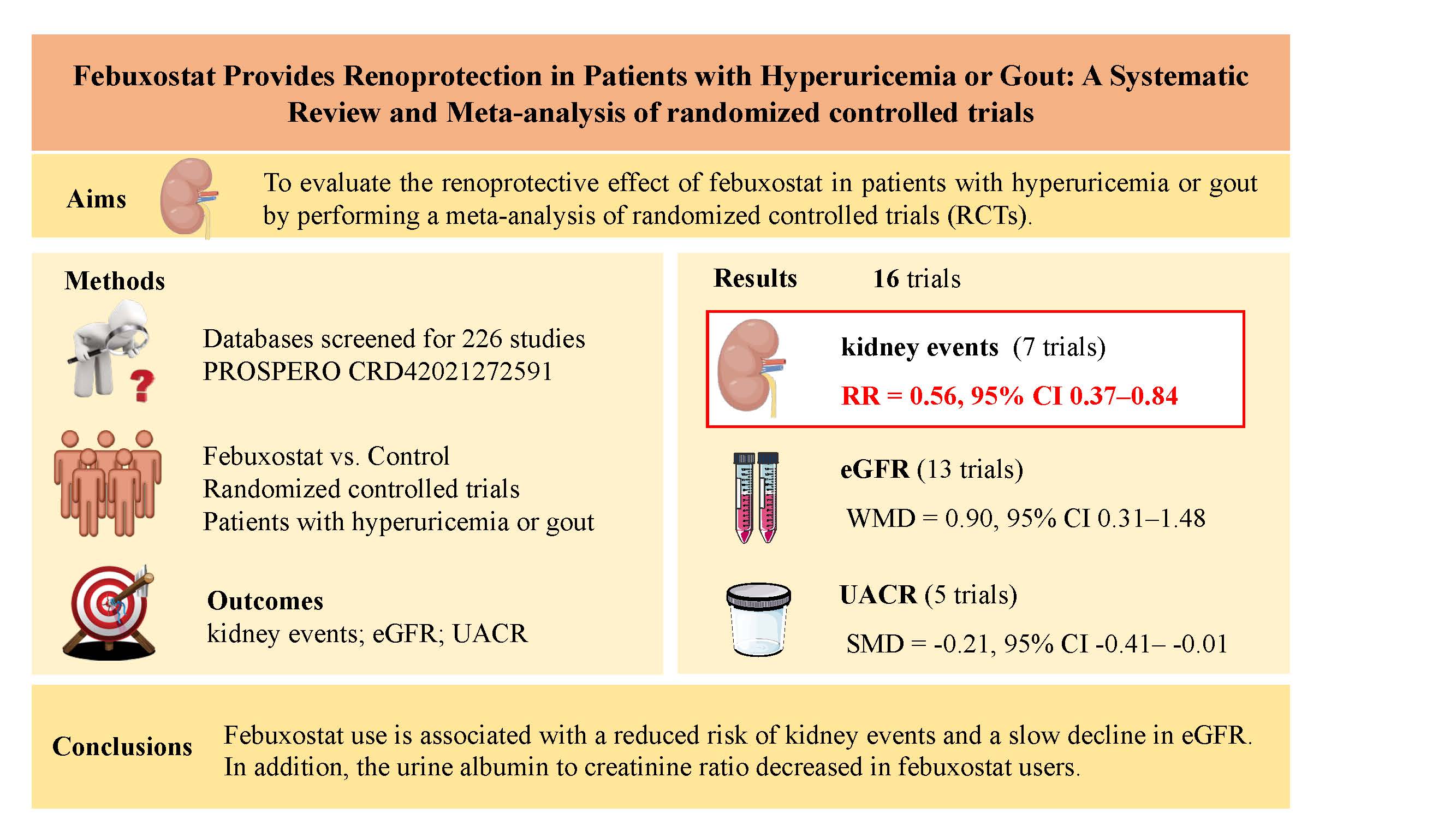

Supplement: Supplemental Material [file IANN_A_2332956_SM8752.zip › graphic/graph_abstract.jpg]
