# Supplementary material for: Febuxostat provides renoprotection in patients with hyperuricemia or gout: a systematic review and meta-analysis of randomized controlled trials
Source: Ann Med. 2024 May 13;56(1):2332956. doi: 10.1080/07853890.2024.2332956 (PMC11095284; doi:10.1080/07853890.2024.2332956)
Supplement: Supplemental Material [file IANN_A_2332956_SM8751.docx]

Table S1: The search strategies of databases

| Databases | Search strategies |
| --- | --- |
| MEDLINE | Search ((("febuxostat, hyperuricemia "[Mesh]) OR ((febuxostat [Title/Abstract])) AND (hyperuricemia [Title/Abstract]) OR uric acid[Title/Abstract]) OR gout [Title/Abstract]) AND randomized controlled trial[Publication Type]) |
| Web of Science | **#1** TS=(febuxostat)  **#2** TS=(hyperuricemia OR uric acid OR gout)  **#3** TS=(randomized controlled trial)  **#4 #3** AND **#2** AND **#1** |
| Embase | **#1** ' febuxostat'/exp  **#2** ' hyperuricemia':ab,ti OR 'uric acid':ab,ti OR 'gout':ab,ti  **#3** 'randomized controlled trial':ab,ti  **#4** #1 AND #2 AND #3 |
| Cocharne library | **#1** MeSH descriptor: [febuxostat] explode all trees  **#2** (hyperuricemia):ti,ab,kw OR (uric acid):ti,ab,kw OR (gout):ti,ab,kw  **#3** (randomized controlled trial):ti,ab,kw  **#4** #1 AND #2 AND #3 |

TableS2: Febuxostat compared to control for kidney events, eGFR and urinary protein or urine albumin creatinine ratio

| **Outcomes** | **№ of participants (studies) Follow-up** | **Certainty of the evidence (GRADE)** | **Relative effect (95% CI)** |
| --- | --- | --- | --- |
|  |  |  |  |
| kidney events | (7 RCT studies) | ⨁⨁⨁⨁ High | RR 0.56 (0.37 to 0.84) |
| eGFR | (13 RCT studies) | ⨁⨁⨁◯ Moderate  Due to serious inconsistency | WMD 0.90 (0.31 to 1.48) |
| urine albumin creatinine ratio | (5 RCT studies) | ⨁⨁⨁◯ Moderate  Due to serious inconsistency | SMD -0.21 (-0.41 to -0.01) |

| CI: confidence interval; RR: risk ratio  GRADE Working Group grades of evidence  High certainty: we are very confident that the true effect lies close to that of the estimate of the effect.  Moderate certainty: we are moderately confident in the effect estimate: the true effect is likely to be close to the estimate of the effect, but there is a possibility that it is substantially different.  Low certainty: our confidence in the effect estimate is limited: the true effect may be substantially different from the estimate of the effect.  Very low certainty: we have very little confidence in the effect estimate: the true effect is likely to be substantially different from the estimate of effect. |
| --- |
